# Experimental Research on the Anti-Reflection Crack Performance of Basalt Fiber Modified Rubber Asphalt Stress-Absorbing Layer

**DOI:** 10.3390/ma17092013

**Published:** 2024-04-25

**Authors:** Cheng Shen, Zhengguang Wu, Peng Xiao, Aihong Kang, Yangbo Wang

**Affiliations:** 1 College of Civil Science and Engineering, Yangzhou University, Yangzhou 225127, China; shenchengyzu@163.com (C.S.); zgwu@yzu.edu.cn (Z.W.); kahyzu@163.com (A.K.); wybyzu@163.com (Y.W.); 2 Research Center for Basalt Fiber Composite Construction Materials, Yangzhou 225127, China

**Keywords:** basalt fibers, rubber asphalt stress-absorbing layer, interlayer bonding performance, anti-reflection crack performance

## Abstract

Reflection cracks are one of the most common problems in semi-rigid base pavement. Setting a stress absorption layer can effectively delay the occurrence of reflection cracks, but further improvement is still needed in its interlayer bonding performance and anti-reflection crack performance. Considering the excellent crack resistance of basalt fibers and the good elastic recovery ability of rubber asphalt, it is considered worthwhile to incorporate them into traditional stress absorption layers to improve performance. To simulate the actual pavement layer effect, composite specimens consisting of a cement-stabilized macadam base + basalt fiber rubber asphalt stress-absorbing layer + AC-20 asphalt mixture surface layer were prepared to evaluate their performance through interlayer direct shear tests, interlayer tensile tests, three-point bending tests, and overlay tests (OTs). To determine the optimal fiber blending combination, four fiber lengths (3 cm, 6 cm, 9 cm, 12 cm) and four fiber proportions (120 g/m^2^, 140 g/m^2^, 160 g/m^2^, 180 g/m^2^) were selected respectively. The specific effects of basalt fibers with different lengths and dosages were analyzed. The results show that compared with the absence of fibers, the improvement of interlayer bonding performance of rubber asphalt with basalt fibers is not significant, and it has certain limitations; however, the improvement of anti-reflective crack performance is significant, with an increase of up to 305.5%. This indicates that the network structure formed by basalt fibers and rubber asphalt stress absorption layer can effectively absorb and disperse external loads, causing an excellent crack resistance effect. Meanwhile, the results indicate that the main factor affecting its interlayer bonding strength and anti-reflective crack performance is the fiber content. Based on the comprehensive analysis of the performance and economy of the stress absorption layer of basalt fiber rubber asphalt, the optimal fiber parameter combination recommended is as fiber length 9 cm and fiber content 160 g/m^2^. These results can provide a reference for the design and performance evaluation of basalt fiber rubber asphalt stress absorption layer, and have certain application value.

## 1. Introduction

Most Chinese high-grade highways use semi-rigid base asphalt pavement [1,2], which is extensively used because of its high overall stiffness, high compressive strength, and good load diffusion capacity, but at the same time, its lack of crack resistance cannot be ignored [3]. Generally, the semi-rigid base is prone to dry shrinkage and temperature shrinkage cracks, which easily transfer upward and expand to the asphalt pavement to form reflective cracks [3,4,5], which greatly reduce the pavement performance and durability. On the one hand, it damages the integrity and continuity of the pavement structure, reduces the overall smoothness of the pavement, and affects the service quality of the pavement; on the other hand, rainwater can continuously seep into the cracks and accumulate in the base layer. Due to the particularity of semi-rigid foundation material, rainwater cannot seep down and stay between the surface layer and the base layer. Under repeated vehicle load, the dynamic water pressure generated by it causes the base to be continuously scoured, weakening the adhesion between the surface layer and the base layer, thus reducing the stability of the pavement.

In order to prevent the early fracturing of asphalt pavement due to cracks in semi-rigid bases, scientists at home and abroad have put forward various anti-reflection crack measures [6,7,8,9,10], and paving modified asphalt fiber stress absorption layer [11,12] has been an effective method. Firstly, because the stress-absorbing-layer [13,14,15] itself had a certain anti-reflective crack effect. Secondly, by adding modified asphalt, the bonding performance between layers can be further improved, and the small deformation energy generated by the base layer can be elastically restored. The addition of fibers [16,17,18] can produce a significant crack resistance effect. The specific reason was because the stress-absorbing layer [19,20] had high stress-absorbing performance, which can prevent the reflection cracking of the semi-rigid base from cracking upwards. It also possessed excellent waterproof performance, preventing rainwater from infiltrating along the crack and damaging the base and good bonding performance, increasing the bonding performance of base and surface course and improving the integrity and bearing capacity. According to the anti-reflection crack mechanism of the stress-absorbing layer, it is proposed that the stress-absorbing layer must have the functional requirements of high elasticity, low temperature flexibility, water damage resistance, and interlayer bonding [21,22]. The fibers are added to the asphalt layer of the stress-absorbing layer. When the force is transferred from the asphalt to the fibers, the deformation of the fibers needs to consume some energy. Once the asphalt is stretched and broken, the fibers will connect to the crack surface. Because the fibers has a strong tensile capacity, it can more effectively prevent or slow down the propagation of cracks [22,23,24].

Basalt fiber is a kind of inorganic high-performance fiber material gradually rising in popularity in recent years. Because of its excellent mechanical properties, stable material performance, and wide distribution of raw materials, it has received more and more attention in asphalt pavement construction [25,26,27,28]. At this stage, scholars at home and abroad have conducted a lot of research on the performance of freshly mixed asphalt mixture at all levels and found that adding basalt fibers into the asphalt mixture can comprehensively improve the road performance of asphalt concrete pavement, such as fatigue cracking resistance, rutting resistance, low-temperature cracking resistance, and water damage resistance [29,30,31], but the research on using basalt fibers in the stress absorption layer has just started.

The main purpose of this study is to develop a type of basalt fiber-modified stress-absorbing layer and subsequently investigate its interlaminar shear resistance, reflective crack resistance, etc. by interlaminar direct shear test, interlaminar pull-out test, three-point bending test, and overlay test (OT). The influence of different fiber parameters on the performance of the stress-absorbing layer is comprehensively compared, and the best basalt fiber parameter combination is selected. It is of great significance to the development of stress-absorbing layer technology and pavement overlay.

## 2. Raw Material Performance Test and Mix Proportion Composition Design

### 2.1. Raw Material Performance Test

The raw materials used in this paper include coarse and fine aggregates, mineral powder, rubber asphalt, basalt fibers, etc. According to the performance requirements of relevant test raw materials, they are tested according to the test methods specified in the test procedures. The test results of all raw materials used in the test meet the requirements of the current specifications. See Table 1, Table 2, Table 3, Table 4 and Table 5 for the specific performance test results.

### 2.2. Mix Proportion Design and Composite Specimen Forming

Considering the particularity of the position of the stress absorption layer, which is located between the surface layer and the base layer, the base layer is usually a cement-stabilized crushed stone base layer, and the surface layer is usually the AC-20 mixture of the lower layer. Therefore, the overall mix design is divided into three parts: the mix design of the cement-stabilized crushed stone base layer, the AC-20 asphalt mixture, and the design of the basalt fiber rubber asphalt stress absorption layer.

#### 2.2.1. Mix Proportion Design of Cement-Stabilized Macadam Base

**Figure 1:**
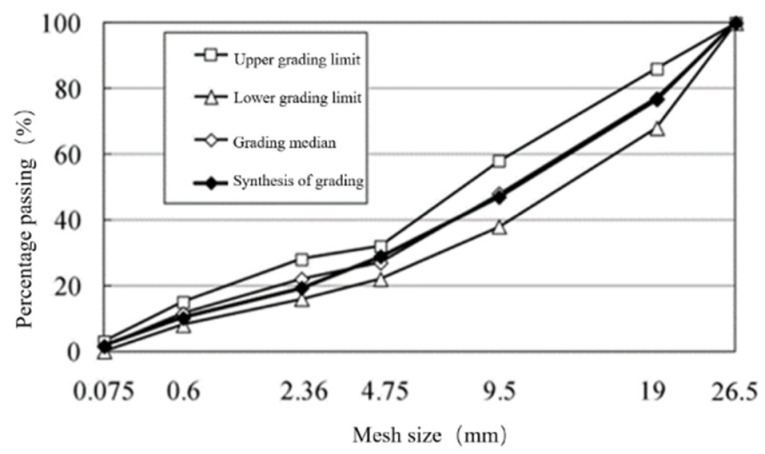
Grading curve of cement-stabilized macadam base.

The aggregate grading type of cement-stabilized macadam mixture is skeleton dense grading (C-B-3) recommended by the current specification technical rules for construction of highway pavement base (JTG/T F20-2015). The composite gradation of cement-stabilized crushed stone mixture is: 1 #:2 #:3 #:4 # =10:29:33:28, and the cement content is 5%. The composite grading curve of cement-stabilized crushed stone base is shown in Figure 1.

Calculate the unconfined compressive strength of the specimen according to the formula. The strength of the cement-stabilized crushed stone base mixture meets the 7-day unconfined compressive strength standard of 4.0~6.0 MPa for cement-stabilized materials used in super heavy traffic road base of Class II and below highways, with a guarantee rate of 95%, which meets the specification requirements.

#### 2.2.2. Mix Proportion Design of AC-20 Asphalt Mixture Surface Course

**Figure 2:**
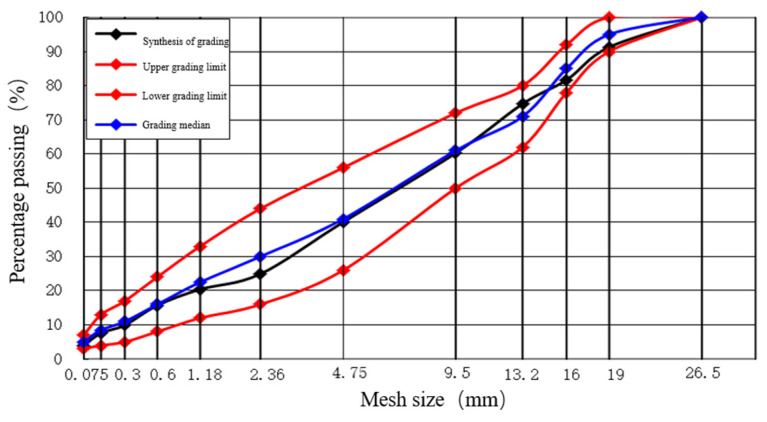
AC-20 asphalt mixture surface gradation curve.

An AC-20 asphalt mixture surface layer is equipped with 4.75 mm as the key sieve, the coarse mixture with the key sieve passing rate less than 45%, and the initially proposed asphalt aggregate ratio is 4.4%. The composite grading of the mixture is 1 #:2 #:3 #:4 #:mineral powder = 23:32:13:29:3. The composite grading curve of AC-20 asphalt mixture surface layer and the upper, lower, and median curves of the grading range are shown in Figure 2.

At the same time, the standard Marshall test method is used to determine the best asphalt aggregate ratio of the AC-20 asphalt mixture. According to the asphalt consumption corresponding to the maximum value of Marshall stability, gross bulk density, and void fraction, the average value of asphalt consumption corresponding to the median value of asphalt saturation oac1 is 4.4%. Therefore, considering the factors of temperature and rutting, the optimum asphalt dosage (OAC) is selected as 4.4%.

#### 2.2.3. Preparation of Basalt Fiber Rubber Asphalt Stress-Absorbing Layer Composite Board

Considering the comprehensive factors such as interlayer performance test and anti-reflection crack performance test, and because the spread gravel is a single-layer gravel stress absorption layer, the gravel particle size range is 4.75–9.5 mm, and the spread amount of gravel is 14 kg/m^2^.The selection range of rubber asphalt dosage is 2.2–2.4 kg/m^2^, and an average value of 2.3 kg/m^2^ is selected in this paper.

According to the specific requirements of the later test on the test piece, 300 mm is uniformly used for the test × 300 mm × 100 mm large rut plate forming. According to the requirements of layered pouring and molding, due to the different height of each layer, the height shall be raised separately. Firstly, mix water, cement, and graded crushed stone according to the measured ratio, and the mixing time is two minutes. After the mixture is fully mixed and uniform, put the mixture into a mold, using the static pressure molding method to form a cement-stabilized crushed stone layer with a thickness of 25 mm. After 24 h, remove the mold and conduct surface polishing treatment and send it to a standard curing room for standard curing for 28 days.

**Figure 3:**
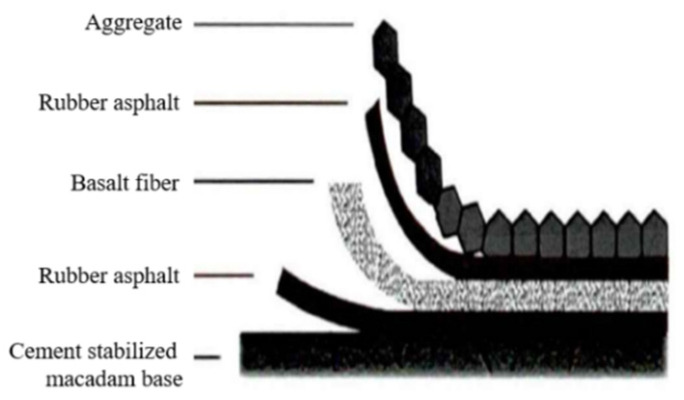
Paving of basalt fiber rubber asphalt stress absorption layer.

Secondly, the basalt fiber rubber asphalt stress-absorbing layer studied is paved onto the cement-stabilized gravel layer under standard curing. After screening, the crushed stone with particle size ranging from 4.75 mm to 9.5 mm is added, with 70 # base asphalt at 0.3% of asphalt to stone ratio for premixing, so as to effectively enhance its adhesion with rubber asphalt. At the same time, the total amount of each fiber is divided into three equal parts. After the first layer of rubber asphalt is distributed, the basalt fibers is distributed in two-dimensional random direction. Generally, the preparation method of rubber asphalt, basalt fibers, rubber asphalt, and ready-mixed crushed stone from the bottom to the top is followed and finally rolled, as shown in Figure 3. In order to ensure that the climbing height of asphalt is 2/3 of the aggregate height and the overall coverage rate in the stress-absorbing layer reaches 96%, three iterations of positive and negative rolling are adopted for rolling formation.

**Figure 4:**
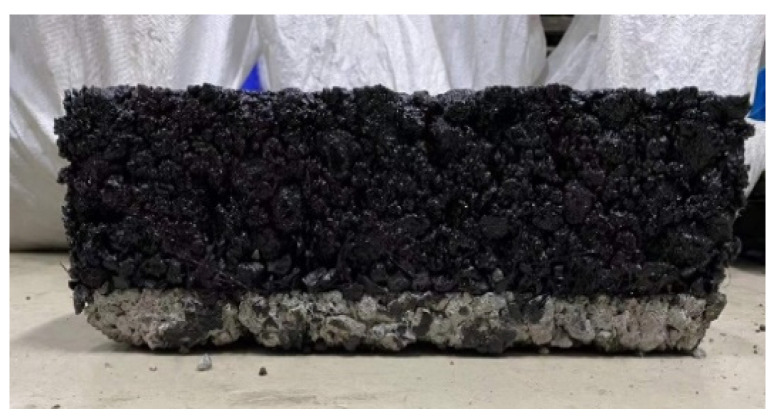
Basalt fiber rubber asphalt stress absorption layer specimen.

Finally, add 60 mm AC-20 asphalt mixture surface layer above the stress absorption layer, use QCX-4 produced by Jingu Shenjian Company in Beijing, China portable combined rut sample forming machine for rolling and molding, and after cooling for 24 h, conduct demolding treatment. Finally, the whole basalt fiber rubber asphalt stress absorption layer composite plate is formed, as shown in Figure 4.

## 3. Test Methods

The interlayer performance of the stress-absorbing layer ensures good coupling between the asphalt layer and the base, improves the service life of the asphalt pavement, and reduces the possibility of interlayer sliding and fracture. It can effectively reduce the interlaminar tensile stress and strain caused by the change of load and moving temperature, and also reduce the possibility of interlaminar slip, so that the overall structural strength of asphalt pavement is enhanced.

The anti-reflective crack performance of the stress-absorbing layer can effectively solve the problem that the semi-rigid base is prone to dry shrinkage cracks and low-temperature shrinkage cracks, and the cracks expand to the asphalt pavement to form reflective cracks under the coupling effect of traffic load and temperature load. The anti-reflection crack ability of the stress absorption layer can effectively inhibit and delay the upward transmission of this kind of reflection crack, and also has the effect of stress absorption.

**Figure 5:**
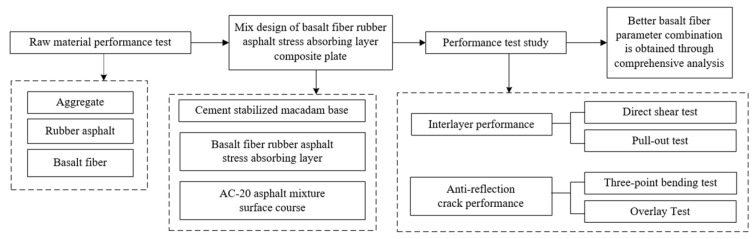
Overall test flow chart.

In this paper, the interlaminar properties of stress absorption are tested by interlaminar direct shear test and interlaminar pull-out test. The anti-reflective crack performance of the stress-absorbing layer is tested by three-point bending test and OT. The overall test process is shown in Figure 5.

### 3.1. Raw Material Performance Test

The maximum shear stress that a bonding material can bear between layers is called the interlaminar shear strength, which is usually used to evaluate its interlaminar shear resistance. In this paper, the direct shear test is used to test the shear resistance between stress absorption layers [32]. The direct shear test maintains the position of the specimen by fixing the fixture, and directly loads the load onto the failure surface of the specimen, so as to test its maximum failure load. Through formula calculation, the corresponding interlaminar shear strength is obtained.

**Figure 6:**
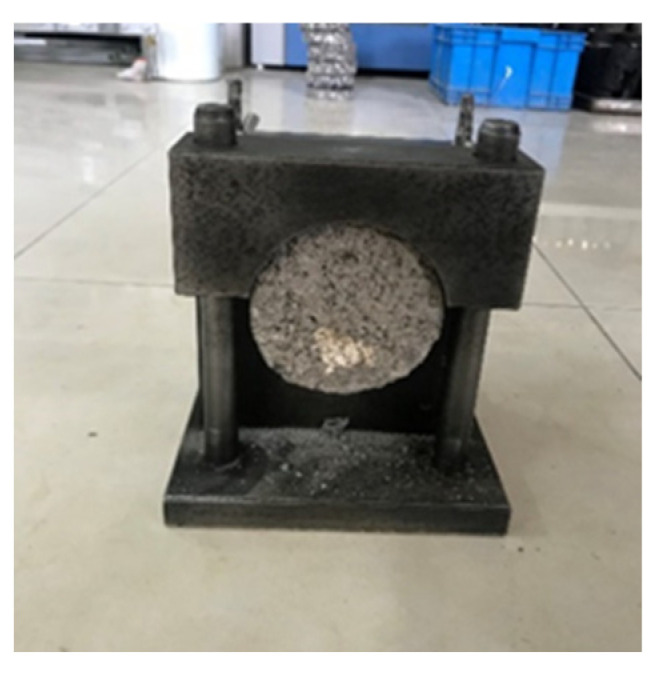
Fixing method of interlaminar shear test mold.

**Figure 7:**
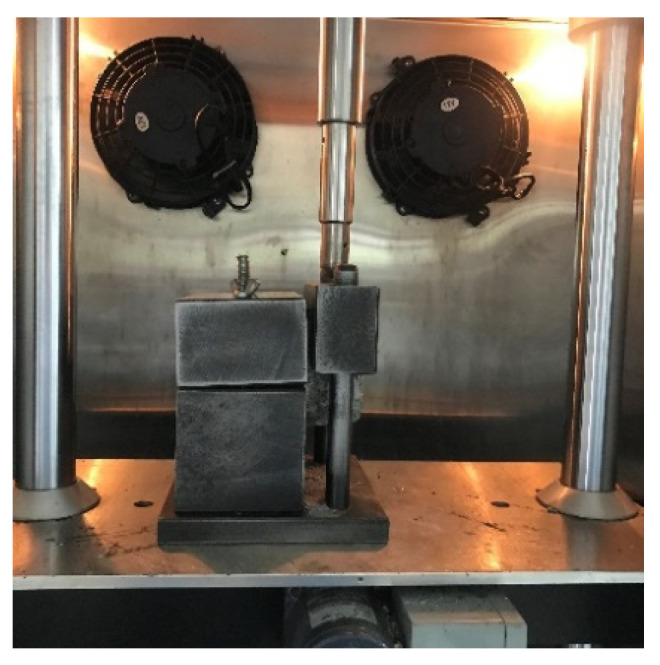
Loading mode of interlaminar shear test.

The specimen is obtained by drilling the core of basalt fiber rubber asphalt stress absorption layer composite plate. The specific size is a cylinder 100 mm in diameter and 100 mm high. During the test, one end of the test piece is fixed with two semicircular clamps, and the other half of the circular clamp is placed above the test piece through the iron bar to undertake the shear force transmitted by the UTM machine, as shown in Figure 6 and Figure 7.

In this test, 25 °C was selected as the test environment to study the interlayer bonding performance. To ensure uniform heating of the test piece, the test piece shall be placed in an oven at the specified temperature for more than 4 h before the interlaminar shear test. Combined with the actual factors, the fast loading mode is adopted, and the loading rate is set to 50 mm/min.

Conduct the interlaminar direct shear test under set conditions until failure. According to the load peak value obtained by the software, the interlaminar shear strength of the stress absorption layer is obtained through Formula (1), which is used as an index to evaluate the interlaminar shear strength of the stress absorption layer.

(1) $$T=\frac{F/1000}{S}$$

In the formula: *T*—interlaminar shear strength, MPa; *F*—peak load, KN; *S*—shear area; for 100 mm diameter test pieces, the contact area between layers is 0.00785 m^2^.

### 3.2. Interlayer Pull-Out Test

The semi-rigid base course easily produces load-type reflection cracks and temperature-type reflection cracks due to its own material characteristics. If the stress absorption layer has good bonding performance, it can reduce the tensile stress and shear stress caused by temperature shrinkage and driving load. The adhesion performance of interlayer materials can be evaluated by the pull-out strength measured by the pull-out tester of pavement interlayer adhesion [33,34].

**Figure 8:**
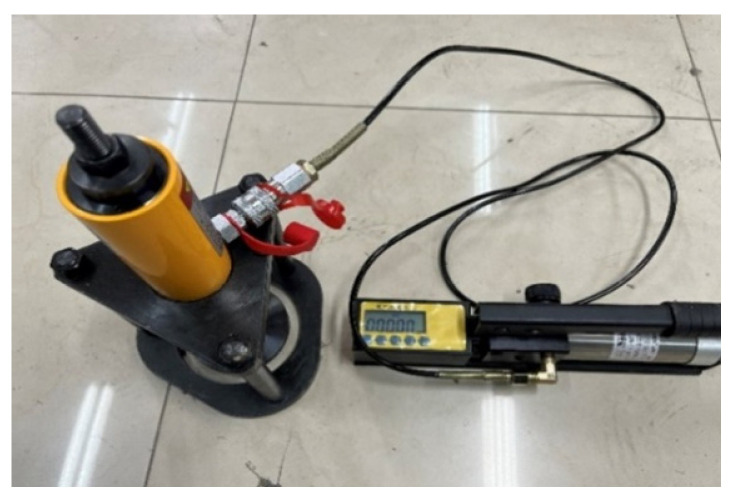
XH-15 T subgrade and pavement adhesion performance tester.

Drill the core of the prepared composite plate test piece. The thickness of the drill core is the thickness of the asphalt mixture, which is 6 cm. One end of the test piece is bonded with a steel plate under the oil cylinder with epoxy resin. After the epoxy resin forms the bonding strength, the test piece is insulated. The XH-15 T produced by Beijing Shengshi Weiye Technology Co., Ltd. in Beijing, China subgrade and pavement bonding performance tester is used to carry out the pull-out test on the composite test piece, as shown in Figure 8. 

**Figure 9:**
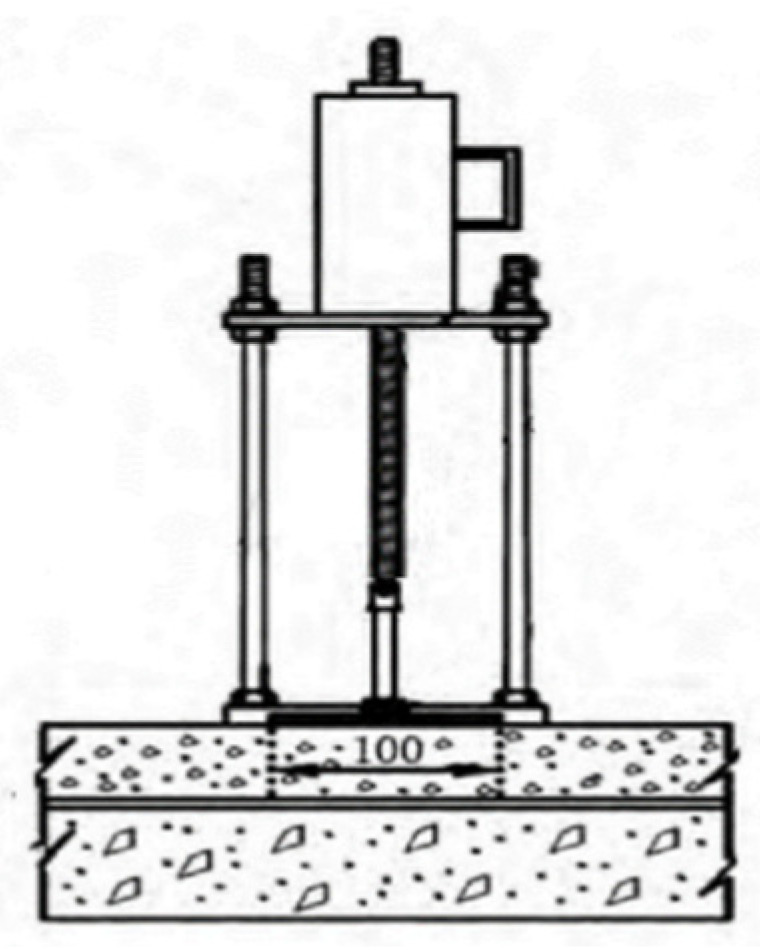
Schematic diagram of drawing test (unit: mm).

During the test, one oil circuit is connected to a pressure sensor and the other is connected with the jack to drive the pull rod to apply tension to the test piece. As the tension gradually increases, when the test piece is stripped, because the pressure on the sensor is equal to the pressure on the jack, the maximum oil pressure at the moment of stripping of the test block can be read out through the digital display circuit. The schematic diagram of pull-out test is shown in Figure 9.

In this test, 25 °C is selected to represent the normal temperature environment. In order to ensure the uniform heating of the test piece, the test piece is kept warm for more than 4 h. During the test, tensile force is applied to the bonding end of the test piece until the test piece is damaged, and the maximum tensile force when it is damaged is measured.

Under the set conditions, apply tension to the bonding end of the specimen until the specimen is destroyed, measure the maximum tension when it is destroyed, calculate the pull-out bonding strength according to Formula (2), and select the pull-out strength as the index to evaluate the bonding performance between layers of basalt fiber stress absorption.

(2) $$\sigma =\frac{F}{S}$$

In the formula: σ—interlayer bonding strength, MPa; *F*—peak load, KN; *S*—shear area; for 100 mm diameter test pieces, the contact area between layers is 0.00785 m^2^.

### 3.3. Three-Point Bending Test

The three-point bending test consists of placing the test piece on two fixed supports with a certain distance and applying a downward load to the test piece along the midpoint direction of the two fixed supports [28]. The specific size is 300 mm long, 100 mm wide, and 100 mm high. A 5 mm crack is pre-cut in the water-stabilized gravel span to ensure that the test piece starts to expand from the tip of the pre-cut crack.

**Figure 10:**
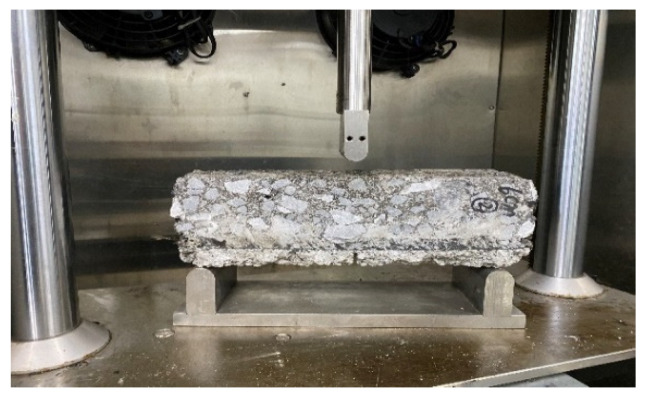
Three-point bending test loading die.

**Figure 11:**
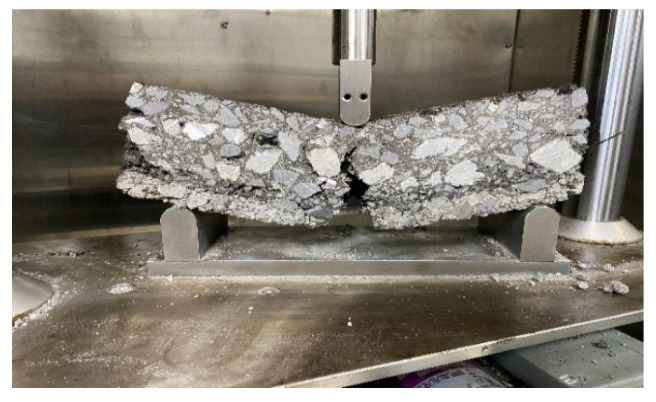
Failure of test piece.

The test piece has three contact points with the support and the compression device, as shown in Figure 10. When two equal cracks are formed, the specimen will break at the midpoint; that is, it will form a three-point bend, as shown in Figure 11, enabling us to measure its disturbance, bending force, and other indicators.

The external force acting on the three-point bending test piece consists of three parts: the work carried out by the load applied to the test piece, the work carried out by the weight *m*_1_ of the beam between the supports, and the work carried out by the weight *m*_2_ of the loading part. Before loading, the force *F*_1_ on the beam is strong, which can be calculated by Formula (3).

(3) $$F_{1}=\frac{1}{2}m_{1}{g+m}_{2}g$$

In the formula: *g*—gravitational acceleration.

**Figure 12:**
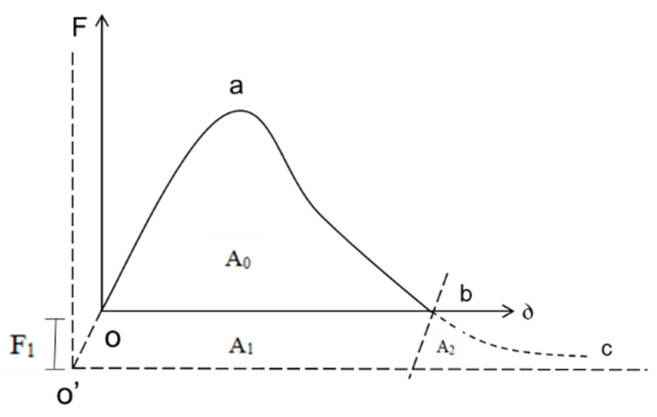
Load deflection curve of three-point bending.

The measured load deflection curve of the test piece is O’ab. When considering the self-weight of the test piece and the weight of the loading part, the load deflection curve is O’ac, as shown in Figure 12.

It can be seen from the figure that the total energy absorbed by the fracture surface of the test piece is *A*; see Formula (4).

(4) $${A=A}_{0}+A_{1}+A_{2}$$

In the formula, *A*_0_ is the area under curve O’ab; see Formula (5) for *A*_1_ and *A*_2_.

(5) $$A_{1}{=A}_{2}=F_{1}\delta_{0}=\left[\frac{1}{2}m_{1}{g+m}_{2}g\right]\delta_{0}$$

The fracture energy $G_{F}$ specified in the test standard can be calculated by Formula (6).

(6) $$G_{F}=\frac{A_{0}+{\mathrm{mg}\delta }_{0}}{A_{\mathrm{lig}}}$$

In the formula, *m* = *m*_1_ + 2*m*_2_, *A_lig_* is the stress area.

A three-point bending die with a mid-span length of 250 mm was used in the test. A temperature of 15 °C is selected for the test to represent the conventional environment. In order to ensure that the test piece is heated evenly, the test piece is placed in an oven at the specified temperature for more than 4 h. Fast loading mode is adopted, and the loading rate is set to 50 mm/min.

According to the requirements of the three-point bending test specification, when a concentrated load is applied to the midspan section of the test piece at the specified rate, when the test piece breaks in the midspan, it is considered that the test piece is damaged, as shown in Figure 11. The data acquisition system records the load–disturbance curve in real time, and the data system provides the maximum bending load, maximum deformation disturbance, and other data results.

### 3.4. Overlay Test

**Figure 13:**
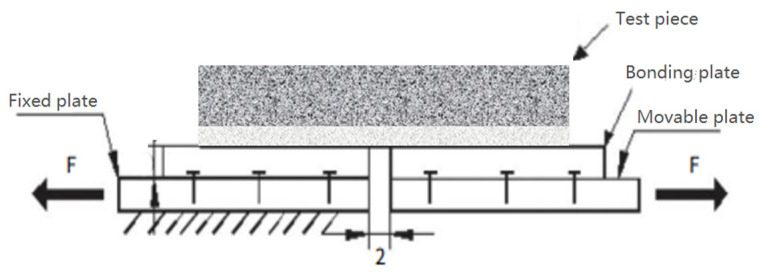
Schematic diagram of overlay test mold.

The three-point overlay test (OT) simulates the tension of asphalt pavement under the test, tests the real impact of reflection cracks on the pavement surface, and can readily evaluate the anti-reflection crack ability of the stress absorption layer [35,36,37]. As shown in Figure 13, the whole test mold is composed of a fixed plate, movable plate, bonding plate, and fixed rivet.

**Figure 14:**
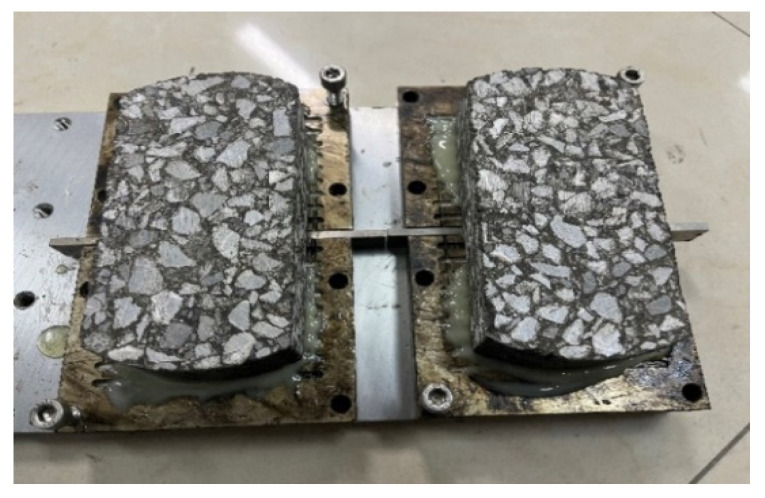
Epoxy resin fixed test piece.

The specific size of the test piece is a length of 150 mm, width of 76 mm, and height of 38 ± 0.5 mm. The prepared OT specimen is bonded to two movable plates with epoxy resin, and a 10 kg weight is placed on each specimen to ensure its firmness under epoxy resin bonding, as shown in Figure 14. After standing and pressurizing for more than eight hours at room temperature, when the bonding strength is sufficient, take the test piece out of the mold.

**Figure 15:**
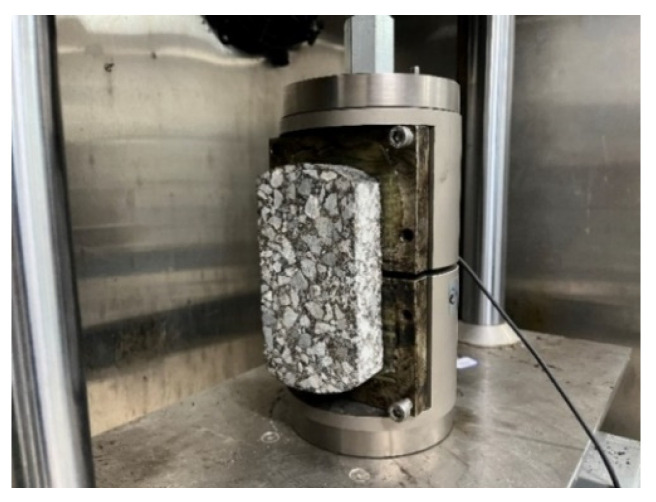
OT loading mold and method.

The test mold is compared to the OT loading mold as shown in Figure 15. There is a gap of 2–3 mm in the middle of the mold. The lower part of the mold is fixed, and the upper part is stretched upward, simulating the effect of a lower reflection crack on the stress absorption layer.

In the test, 25 °C is selected to represent the normal temperature environment. In order to ensure that the test piece is heated evenly, the test piece is placed in an oven at the specified temperature for more than 4 h. The test load loading waveform adopts triangular wave, and the load cycle is 10 s. The data acquisition system records the cycle–displacement–load curve in real time, and the data system provides data results such as the number of test cycles, load loss rate, the maximum load value of the first cycle, etc.

According to the requirements of OT specification [20], taking the length of crack displacement as the control index, the target displacement value of the crack is 0.635 mm, and the closing movement of the reflection crack is simulated through the above parameters. When the maximum load of the specimen in a certain cycle decreases by more than 93% compared with the maximum load of the initial cycle, it is considered that the specimen is damaged and the test will stop automatically. At the same time, the test operation cycle is set as 1000 iterations in the specification. When the actual operation cycle is reached, the system will also recognize that the test has stopped automatically.

In OT, there are two different stages of cracking, namely, the crack initiation stage and the crack propagation stage.

The fracture energy at the crack initiation stage is calculated by Formula (7).

(7) $$G_{A}=\frac{1}{\mathrm{tb}}\int_{W_{1}}^{W_{2}}f(w)\mathrm{dw}$$

The fracture energy at the crack growth stage is calculated by Formula (8).

(8) $$G_{B}=\frac{1}{\mathrm{tb}}\int_{W_{2}}^{W_{3}}f(w)\mathrm{dw}$$

Therefore, the calculation formula of total fracture energy is shown in Formula (9).

(9) $$G_{f}{=G}_{A}+G_{B}=\frac{1}{\mathrm{tb}}\int_{W_{1}}^{W_{3}}f(w)\mathrm{dw}$$

In the formula: F(w)—load–displacement curve; *t*, *b*—thickness and width of test specimen; *W*_1_, *W*_2_, *W*_3_—different displacements corresponding to load–displacement curve.

## 4. Test Results and Analysis

### 4.1. Interlaminar Direct Shear Test

**Figure 16:**
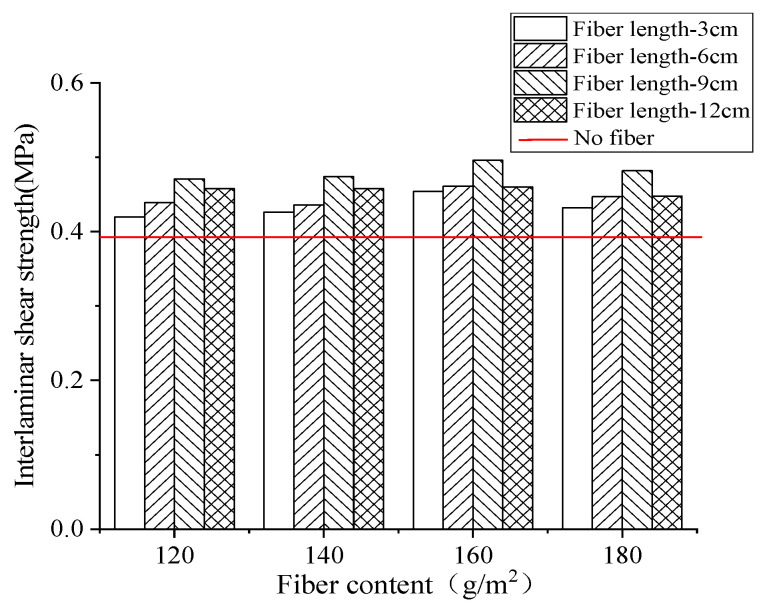
Effect of fiber content on interlaminar shear strength.

The effect of different fiber contents on interlaminar shear strength is shown in Figure 16.

As can be seen from Figure 16, the interlaminar direct shear strength without adding fibers was 0.396 MPa. Compared with the control group without fibers, when the fiber content was 160 g/m^2^, the interlaminar shear strength of the basalt fiber rubber asphalt stress-absorbing layer with a length of 3 cm, 6 cm, 9 cm, and 12 cm was increased by 14.6%, 16.4%, 25.3%, and 16.1%, respectively. Compared with other dosage groups, its improvement effect was better.

It can be seen from Figure 16 that when the fiber content was 160 g/m^2^, the interlaminar shear strength of the basalt fiber rubber asphalt stress-absorbing layer with the length of 3 cm, 6 cm, 9 cm, and 12 cm increased by 14.6%, 16.4%, 25.3%, and 16.1%, respectively, compared with other content, which was better than other content.

**Figure 17:**
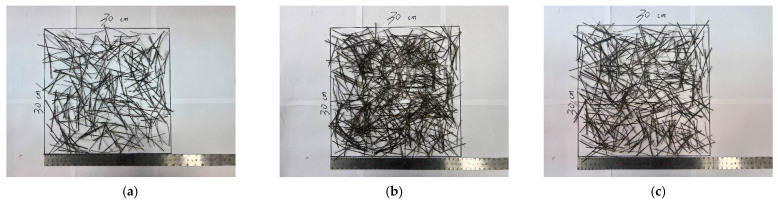
(**a**) Insufficient fibers leads to sparsity. (**b**) Excessive fibers leads to overlap. (**c**) Uniform distribution of fibers.

The interlaminar shear strength of rubber asphalt stress-absorbing layer with different basalt fiber content increased at first and then decreased with the increase of content. This was because the increase of the content of the mixture led to the gradual reduction of the interlayer porosity, which made the upper and lower bond of the asphalt layer firmer and increased the interlayer shear strength. However, when the fiber content was increased to 180 g/m^2^, in the case of uniformly distributed fibers, the fibers were surplus, and there was a lot of overlap between the fibers, which made the asphalt layer at the overlap develop delamination, thus reducing the interlaminar shear strength. The three situations of sparse, uniform, and excessive fiber distribution are shown in Figure 17.

In general, when the fiber length was 9 cm and the fiber content was 160 g/m^2^, the interlaminar shear resistance of the rubber asphalt stress absorption layer was the strongest. This was because the fiber length was compatible with the gravel particle size, and the optimal fiber length was closely related to the maximum nominal particle size. When the particle size of crushed stone was 4.75~9.5 mm, with the increase of fiber length, the number of crushed stones that can be completely connected by the fibers gradually increases, making the shear resistance gradually improve. When the fiber length was 9 cm, the number of crushed stones that could be connected was the largest, and the compatibility with crushed stones within the particle size range was the highest. When the stress-absorbing layer was sheared, the maximum shear stress could be borne.

**Figure 18:**
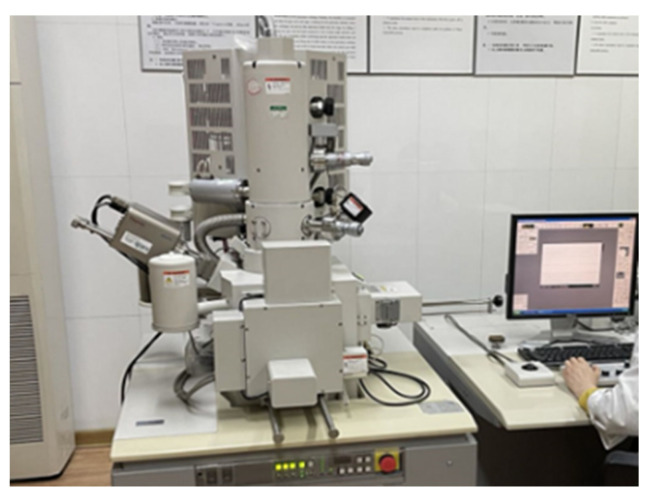
Scanning electron microscope.

**Figure 19:**
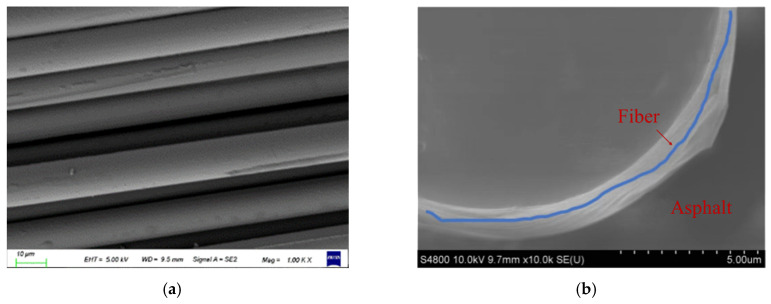
Microscopic morphology of basalt fibers: (**a**) Basalt fibers. (**b**) Basalt fiber–rubber asphalt.

We used a scanning electron microscope (as shown in Figure 18) to take photos of the micro-morphology of the fibers, and at the same time, we took photos of the micro-morphology observed under the contact conditions between the fibers and asphalt binder. We analyzed the surface micro-morphology characteristics, and the micro-morphology image is shown in Figure 19.

At the micro scale, scanning electron microscopy experiments can help reveal the true state of the fibers. From Figure 19, it can be seen that the short-cut basalt fibers do not undergo bending and winding and can form a good bridging effect in the mixture. The flocculent basalt fibers were cotton-like fibers formed by bending basalt fibers, which helped to form a three-dimensional network structure, allowing asphalt to adsorb to the fiber surface, making the distribution of asphalt slurry between aggregates more stable, thereby increasing the thickness of the asphalt film and improving the bonding strength between asphalt and aggregates. At the same time, the above viewpoint can be verified through microscopic images of fiber asphalt slurry. Both types of basalt fibers and asphalt binders had clear distinguishing areas, which effectively increased the contact surface between fibers and asphalt, generated anchoring effects during the interaction between fibers and asphalt, and further improved the adhesion between fibers and asphalt.

### 4.2. Interlayer Pull-Out Test

**Figure 20:**
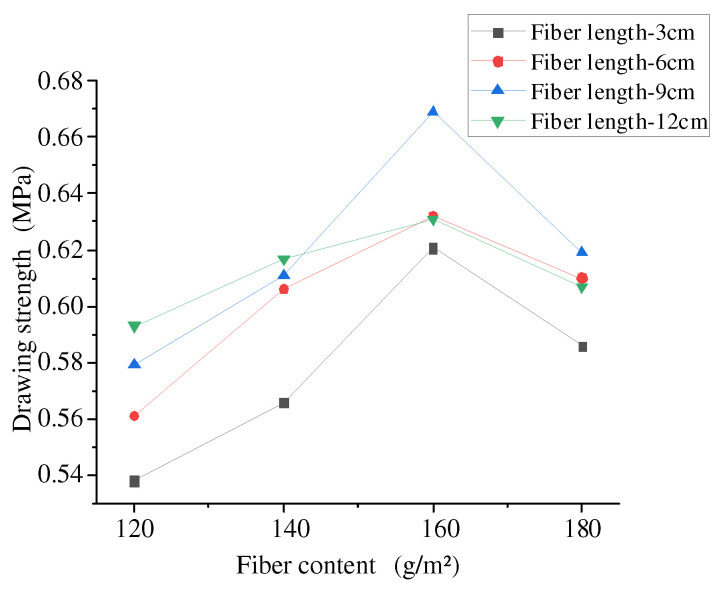
Effect of fiber content on drawing strength.

The effect of different fiber contents on the bonding performance between stress-absorbing layers is shown in Figure 20.

The experimental data showed that the interlaminar tensile strength without fiber incorporation was 0.312 MPa. Compared with the blank group without fiber incorporation, with fiber incorporation, fiber combinations with different parameters improved interlaminar tensile strength with a significant increase in the range of 72% to 114% and with a significant improvement in tensile strength.

It can be seen from Figure 20 that the influence of different fiber lengths on the pull-out strength of rubber asphalt stress-absorbing layer had certain similarity with the change of the content. The overall curve trend was gradually increasing at first, and when the fiber content was 160 g/m^2^, it reached the peak value at the same length, and then gradually decreased.

When the fiber content was 120 g/m^2^ and 140 g/m^2^, the reinforcement effect of basalt fibers on drawing strength was linear with the increase of fiber length, and increased with the increase of fiber length. When the fiber content was 160 g/m^2^ and 180 g/m^2^, the reinforcement effect of basalt fiber increased first and then decreased with the increase of fiber length, and decreased after the peak value at the fiber length of 9 cm. Different from the results of direct shear test, when the fiber content was 120 g/m^2^ and 140 g/m^2^, the interlaminar tensile strength gradually increased. This was because the fiber content was related to the total contact area of asphalt. When the fiber content was small, the greater the fiber dispersion was under the same spreading area, which encouraged more fibers to make contact with rubber asphalt, absorbed the light components in asphalt, increased the thickness of asphalt film, and thus enhanced the adhesion between layers.

On the whole, compared with the blank group without fibers, when the fiber length was 9 cm and the fiber dosage was 160 g/m^2^, the basalt fibers significantly improved the interlayer bonding performance of the stress absorption layer, because the basalt fibers itself had better asphalt adsorption capacity, and the asphalt could evenly wrap around the fiber surface and produce a physical reaction with it, forming a structural asphalt layer with a stronger bonding force and increasing the interlayer bonding performance.

### 4.3. Three-Point Bending Test

#### 4.3.1. Test Results

**Figure 21:**
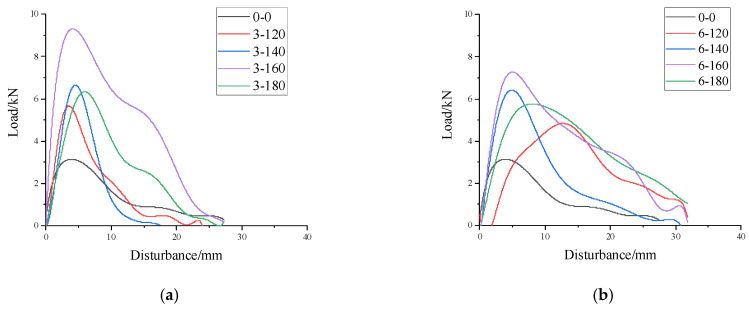

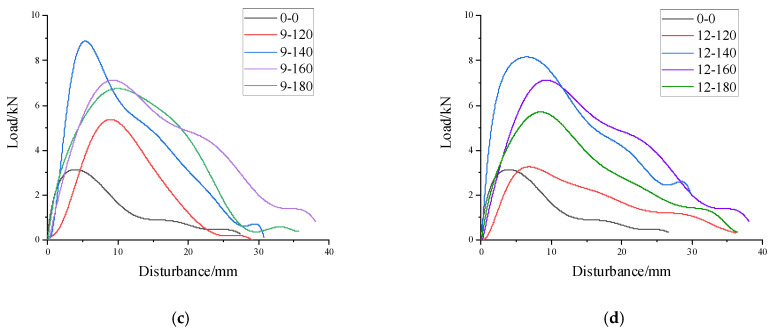
Load-deflection curve: (**a**) 3 cm group; (**b**) 6 cm group; (**c**) 9 cm group; (**d**) 12 cm group.

The test parameters that can be directly read on the test instrument selected in this test were maximum bending load *F* and maximum deformation disturbance *L*. According to the relevant research regarding the three-point bending test, the bending fracture energy *W*_0_ was selected as the evaluation index. The bending fracture energy *W*_0_ was the ratio of the area surrounded by the load disturbance curve of the specimen to the stress area of the specimen. See Figure 21 for different groups of load deflection curves.

#### 4.3.2. Effect of Basalt Fiber on Bending Fracture Energy

**Figure 22:**
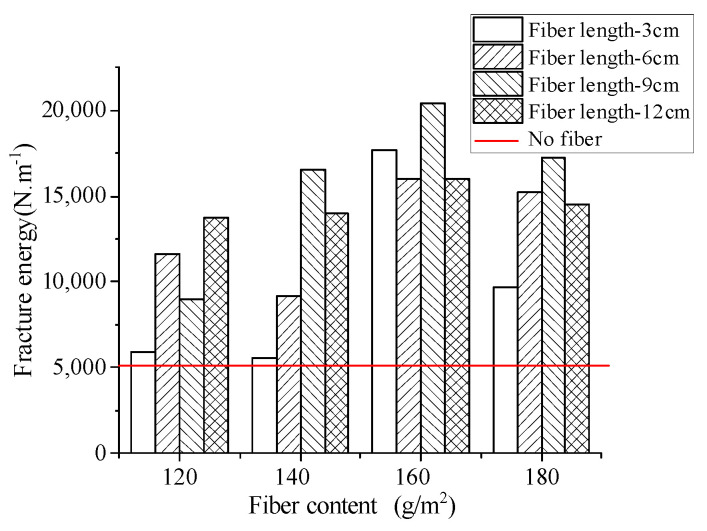
Effect of fiber content on bending fracture energy.

The influence of different fiber contents on the bending fracture energy of the stress-absorbing layer is shown in Figure 22.

As can be seen from Figure 22, the three-point bending fracture energy without fiber incorporation was 5036.7 N·m^−1^. Compared with the blank group without fiber incorporation, with fiber incorporation, fiber combinations with different parameters had a significant improvement in fracture energy, and the increase was significant.

When the fiber content was 160 g/m^2^, the bending fracture energy of the stress-absorbing layer was significantly improved by 249.3%, 218.9%, 305.5%, and 217.6% for rubber asphalt stress-absorbing layers mixed with basalt fibers of 3 cm, 6 cm, 9 cm, and 12 cm length, respectively. In general, when the fiber length was 9 cm and the fiber content was 160 g/m^2^, basalt fibers had the best effect on improving the anti-cracking performance of the stress absorption layer.

With the increase of fiber length, the distribution area of fibers decreased at the same dosage, resulting in fewer fibers at the crack to prevent cracking. According to the observations of the test, when the test piece started to break, the fibers of each length can produce a crack resistance effect at the crack, but with the continuous expansion of the crack, the basalt fibers with the lengths of 3 cm and 6 cm were gradually separated, while the basalt fibers with the length of 9 cm and 12 cm could still be connected at the crack until the test piece was completely broken.

With the increase of fiber content, the bending fracture energy of the rubber asphalt stress-absorbing layer first increased and then decreased. This was because the increase of the fiber content made the fiber distribution density increase, and there were more fibers at the crack to play the role of “reinforcement and crack resistance” [38], but the excessive content reduced the overall orientation of the fiber asphalt layer, reducing the tensile performance of the fiber asphalt layer at the crack.

### 4.4. Overlay Test

#### 4.4.1. Test Results

**Figure 23:**
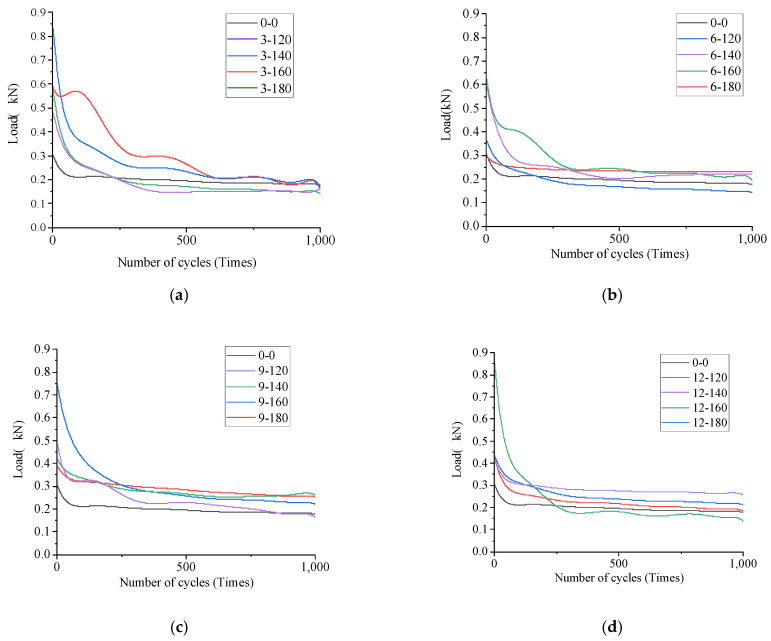
Load-cycle iteration curve: (**a**) 3 cm group; (**b**) 6 cm group; (**c**) 9 cm group; (**d**) 12 cm group.

The test parameters that can be directly read on the test instrument selected in this test were the number of test cycles (*N*), the load loss rate (*R*), and the maximum load (*F*) in the first cycle. At the same time, according to the relevant research of OT, the total fracture energy (*G*) was selected to represent the total fracture energy during cracking, which was used as an index to evaluate the anti-reflection crack performance of the stress absorption layer. The OT load cycle curve under different mix combinations is shown in Figure 23.

**Figure 24:**
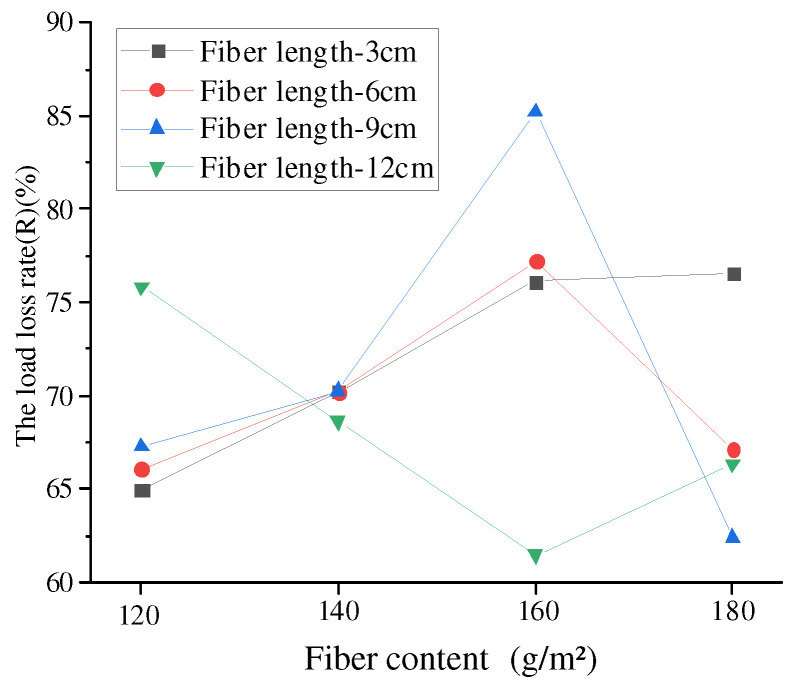
The load loss rate (*R*).

It can be seen from Figure 24 that when the number of test cycles of 17 groups of stress-absorbing layers reached 1000, the load loss rate of the stress-absorbing layer without fibers was the least, and the *R* value was 60.05%. With the increase of fiber length and fiber content, the *R* value mostly shows a trend of rising first and then declining. When the fiber length was 9 cm and the fiber content was 160 g/m^2^, the load loss rate was the largest, and the *R* value was 85.22%, which indicates that in the whole failure cycle of reflection crack, the fiber length was 9 cm. When the fiber content was 160 g/m^2^, the stress-absorbing layer needed the most energy to destroy, and more force and energy were needed to form reflective cracks.

However, the R value of the 12 cm fiber shows a trend of first decreasing and then increasing, because at the same dosage, the excessive fiber length leads to excessive concentration of fatigue cracks and development along the fiber length. The shorter the length of the fiber, the more it can disperse and form a strong network structure during distribution. However, the 12 cm fibers could only form a smaller fiber bundle-like structure at the ends of the fibers, while the middle part of the fiber is still a ball and not effectively separated. However, when the dosage increases to a certain amount, this undivided fiber forms a larger fiber skeleton, similar to the “reinforcement” effect, which can play a certain role in delaying load loss, but the effect is not significant and the improvement is not significant.

#### 4.4.2. Effect of Basalt Fiber on Anti-Reflection Crack Performance

**Figure 25:**
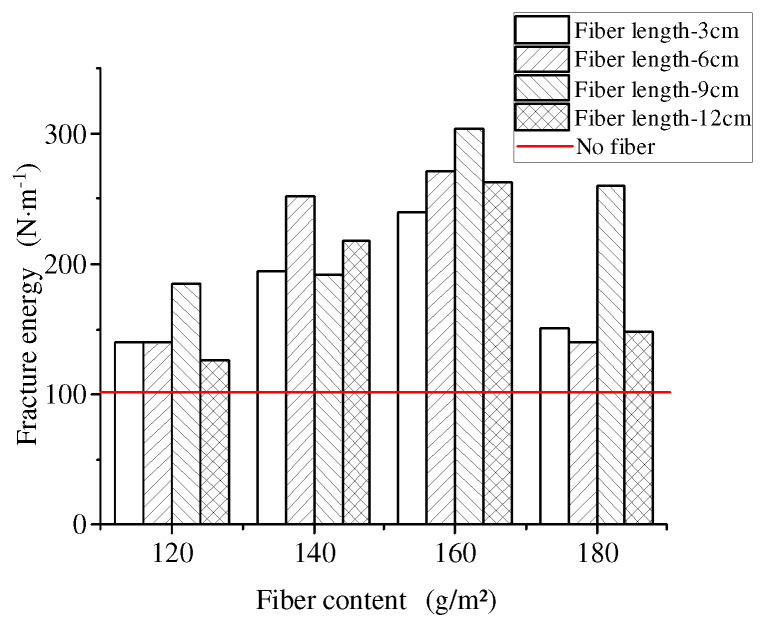
Effect of fiber content on total breaking energy.

The influence of different fiber contents on the anti-reflection crack performance of rubber asphalt stress absorption layer is shown in Figure 25.

It can be seen from Figure 25 that the addition of basalt fibers can significantly improve the anti-reflection crack performance of the stress absorption layer. The total fracture energy of the stress-absorbing layer without fibers was 101.05 N·m^−1^. Under the condition of fixed fiber length, the total breaking energy first increased and then decreased with the increase of fiber content. When the fiber length was 9 cm and the content was 120 g/m^2^, 140 g/m^2^, 160 g/m^2^ and 180 g/m^2^, the total fracture energy of the rubber asphalt stress-absorbing layer increased by 82.3%, 90.31%, 200.26% and 157.3%, respectively.

In general, when the fiber length was 9 cm and the fiber content was 160 g/m^2^, basalt fibers presented the best effect on the anti-reflection crack performance of the stress absorption layer. This was because the increase of fiber content made the stress dissipation rate of rubber asphalt stress absorption layer gradually increased. The faster the stress dissipation rate was, the less likely the stress absorption layer was to crack, but too much fiber content was not conducive to deformation recovery.

On the other hand, when the fiber content was fixed, with the increase of fiber length, the total fracture energy of the rubber asphalt stress absorption layer showed a trend of rising first and then falling. However, when the fiber length was too long to cause fatigue cracks in the specimen, the cracks were excessively concentrated and developed along the fiber length, thus reducing the anti-reflection crack performance. At the same time, this rule was also consistent with the change rule of test R value (load loss rate measured by the system).

#### 4.4.3. OT Test Curve Fitting

According to Figure 24, it can be found that the maximum load cycle curve of 17 basalt fiber stress absorption layers with different length and content under conventional conditions conformed to the change law of power function, so the power function shown in Formula (10) was selected to fit the test curve [39].

(10) $$y=ax^{-b}$$

In the formula, a,b were parameters. Among them, parameter “*a*” represents the decline rate of the curve. The larger its value, the faster the decline rate of the curve. At the same time, it also indicates that the faster the load attenuation rate of the stress absorption layer was, the faster the damage of the stress absorption layer was, and the worse the anti-reflection crack performance was. We fit the OT curve and obtained the fitting results shown in Table 6.

Through the curve fitting results, it was found that the variation law of load cycle curve of basalt fiber stress absorption layer was very consistent with the variation law of power function. It can be seen from Table 6 that the value of the parameter “*a*” of the stress absorption layer without fibers was the largest, reaching 1.824, while the *a* value was the smallest when the fiber dosage was 160 g/m^2^ and the fiber length was 9 cm, reaching 0.6807, which meant that the stress absorption layer without basalt fibers had the fastest destruction speed, the stress absorption layer had the slowest destruction speed when the fiber dosage was 160 g/m^2^ and the fiber length was 9 cm, and the anti-reflection crack performance was the strongest, which was basically consistent with the influence law of the above fiber parameters on the fracture energy.

### 4.5. Comprehensive Engineering Analysis

#### 4.5.1. Engineering Performance Analysis

In this paper, the performance of basalt fiber rubber asphalt stress-absorbing layer was evaluated and analyzed by interlaminar direct shear test, interlaminar pull-out test, three-point bending test and OT. Overall, fiber content played the major affecting role on the performance of rubber asphalt stress absorption layer rather than the fiber length.

The fiber parameter combination with the best improvement effect of fiber length of 9 cm and fiber content of 160 g/m^2^ was selected to analyze the test results of basalt fiber rubber asphalt stress-absorbing layer under four tests. The improvement ranges of different properties under the same content are shown in Table 7.

It can be seen from Table 7 that compared with the blank group without fibers, the properties of the rubber asphalt stress-absorbing layer mixed with basalt fibers had been improved to a certain extent, and the increase of interlaminar shear strength was small, only 25.2%. The increase of bending fracture energy and total fracture energy was obvious, both being greater than 200%, and the improvement effect of bending fracture energy was the best, up to 305.5%. This showed that the basalt fiber combination with fiber length of 9 cm and fiber content of 160 g/m^2^ can significantly enhance the cracking resistance of the rubber asphalt stress absorption layer. This was because the basalt fiber acts as a “bridge” in the cracking process of the specimen, thus effectively preventing or delaying the speed of crack initiation and propagation in the rubber asphalt stress absorption layer.

As the number of cycles increased, the stress drop in the blank sample suddenly rose, and then the pavement became cracked (after about 70 cyclic loading times) [14,20]. In comparison, the rate at which the stress dropped in the test sample with a CSAL was significantly slower and the load decreased smoothly to 7% of the initial value after 1000 cycles. The fatigue life of the sample with the RASAL resisting the formation of reflective cracks was more than 10 times that of the blank sample at this lower temperature. In the same situation, the stress absorption layer of basalt fiber rubber asphalt has less load loss after 1000 cycles of action, which means it has stronger stress absorption effect. If reflection cracks are not to cause damage, a stronger ability is needed.

#### 4.5.2. Engineering Economic Analysis

Taking the asphalt pavement maintenance project of the Lima Expressway in 2022 as an example, a rubber asphalt stress absorption layer was used in both the overlay maintenance plan and the service area square pavement and ramp disposal planed in this major maintenance project. The construction plan design diagram is shown in Table 8. According to the bill of quantities for the 2022 Lima Expressway Asphalt Pavement Overhaul Project, a total of approximately 32,000 m^2^ rubber asphalt stress-absorbing layers were used in this project, with a total contract amount of over CNY 1.4 million. The average unit price of the rubber asphalt stress-absorbing layer during this process was calculated to be approximately CNY 43.5 /m^2^.

From the comparison of raw material composition and dosage, it can be seen that compared with the ordinary rubber asphalt stress absorption layer, the basalt fiber rubber stress absorption layer only added a layer of basalt fiber layer in the middle of the rubber asphalt layer, with a fiber length of 9 cm and a dosage of 160 g/m^2^, while maintaining the same type and dosage of other raw materials. According to the current market price, the selling price of basalt fiber was about CNY 20,000/ton, so the cost of selecting basalt fiber rubber asphalt stress absorption layer was about CNY 47/m^2^. Compared to the unit price of CNY 43.5/m^2^ for ordinary rubber asphalt stress absorption layer, the cost of basalt fiber rubber asphalt stress absorption layer only increased by CNY 3/m^2^. Based on the performance analysis above, it can be seen that the crack resistance and reflection crack resistance of the stress-absorbing layer had been improved by 305.5% and 200.2%, respectively, with significant economic benefits. Therefore, the basalt fiber rubber asphalt stress absorption layer was a low-cost and efficient reflection crack prevention and control measure.

According to relevant engineering research data, compared to pavement structures without a stress absorption layer, laying a rubber asphalt stress absorption layer on the basis of a semi-rigid base will greatly improve the service life of the road structure. By relying on its high-fatigue performance, the overall fatigue extension life of the structure can be increased by 36%. At the same time, the production of rubber powder has become the leading direction for the reuse of waste tires. Vigorously promoting the application of rubber powder and rubber asphalt is not only conducive to promoting the recycling and reuse of resources, achieving the goal of saving natural resources, but also conducive to reducing environmental pollution and improving human living environment.

Therefore, on the one hand, it reflects the construction requirements of Chinese “long life roads”, effectively reducing the frequency of road upgrades and maintenance; on the other hand, it also fundamentally reduces the consumption of natural resources and energy, indirectly playing a role in energy conservation, low-carbon and environmental protection, in line with the current strategic goal of peak carbon and carbon neutrality.

## 5. Conclusions

In this paper, a new type of basalt fiber modified rubber asphalt stress-absorbing layer was prepared. It was applied into the composite plate in the form of a cement-stabilized macadam base + basalt fiber rubber asphalt stress-absorbing layer + AC-20 asphalt mixture surface layer. The interlaminar shear resistance, interlaminar bonding performance and anti-reflection crack performance were investigated, and the influence of basalt fiber length and content on the performance of the absorbing layer was analyzed. The main conclusions can be drawn as follows:

- From the perspective of interlayer bonding performance, compared with the neat samples without fiber addition, the improvement in interlayer bonding strength of basalt fiber modified samples significantly achieves up to 114%, while the improvement of interlayer direct shear strength is not very significant, with a maximum reinforcement amplitude of only 20%. The reinforcement of the addition of basalt fiber mainly reflect in the improvement of the cohesion of asphalt binder rather than the interface strength.
- Based on the three-point bending test, the fracture energy of basalt fiber modified samples increases by 305.5% compared with the neat samples. Basalt fiber can enhance the anti-cracking performance some extent under one-time failure loads. Furthermore, optimal fiber content should be determined since excessive fiber will cause negative effect on the anti-cracking performance.
- Based on the overlay test, the maximum increase in fracture energy of basalt fiber-modified samples can reach up to 200.3%, compared with the neat samples. Basalt fibers possess a significant impact on the anti-reflection cracking performance under cyclic loads. Similarly, optimal fiber content should be determined since the fracture energy first increases and then decreases with an increasing fiber content.
- According to range analysis, the main factor affecting the anti-reflection cracking performance was the fiber content rather than the fiber length.
- Based on comprehensive performance and cost-effectiveness analysis, it was recommended that the basalt fiber parameter combination with the length of 9 cm and fiber content of 160 g/m^2^ can be used to prepare the new type of basalt fiber modified rubber asphalt stress absorption layer.

## Figures and Tables

**Table 1:** Aggregate test results.

Types		Apparent Relative Density	Relative Density of Gross Volume
Limestone	1# (19–13.2 mm)	2.724	2.683
	2# (13.2–2.36 mm)	2.715	2.695
Basalt	3# (4.75–0.15 mm)	2.975	2.888
	4# (0.15–0.075 mm)	2.970	2.863

**Table 2:** Technical indicators of coarse aggregate quality.

Test Index	Test Result	Specification Requirements	Test Method
Crushing value/%	14	≤26	T0316
Los Angeles abrasion value/%	16	≤28	T0317
Apparent relative density/g/m^2^	2.883	≥2.6	T0304
Water absorption/%	0.7	≤2	T0305
Adhesion to asphalt/grade	5	≥4	T0616
Water washing method < 0.075 particle content/%	0.4	≤1	T0310

**Table 3:** Technical indicators of fine aggregate quality.

Test Index	Test Result	Specification Requirements	Test Method
Apparent relative density/g/m^2^	2.762	≥2.5	T0330
Firmness (>0.3)/%	9	≤12	T0340
Sand equivalent/%	66	≥60	T0334

**Table 4:** Performance indicators and test results of rubber asphalt.

Test Index		Test Result	Specification Requirements	Test Method
Penetration (25 °C)/0.1 mm		44	30~60	T0604
Penetration index (PI)		0.8	≥0	T0604
Softening point/°C		68	≮60	T0606
Ductility (5 cm/min, 5 °C)/cm		9	≮5	T0605
Elastic recovery (25 °C)/%		86	≮60	T0662
Segregation (Softening point difference)/°C		1.8	≯3	T0661
Residue after RTFOT	Quality change/%	−0.06	±1.0	T0610
	Penetration ratio/%	76	≮60	T0604
	15 °C residual ductility/cm	28	≮10	T0605

**Table 5:** Performance indexes and test results of basalt fibers.

Test Index	Specification Requirements	Test Result
Elongation at break/%	≤3.1	2.71
Breaking strength/MPa	≥1200	2218
Oil absorption rate/%	≥50	52
Heat resistance, breaking strength retention/%	≥85	93
Alkali resistance, breaking strength retention/%	≥75	89

**Table 6:** Fitting results of OT curve.

Length (cm)–Content (g/m^2^)	Fitting Formula	*R* ^2^	*a*	*b*
0–0	$y=1.8246x^{-0.6364}$	0.9783	1.8246	0.6364
3–120	$y=1.364x^{-0.4644}$	0.9657	1.364	0.4644
3–140	$y=1.266x^{-0.4732}$	0.9816	1.266	0.4732
3–160	$y=0.8547x^{-0.3558}$	0.976	0.8547	0.3558
3–180	$y=1.343x^{-0.419}$	0.9872	1.343	0.419
6–120	$y=1.2139x^{-0.4738}$	0.9693	1.2139	0.4738
6–140	$y=1.4131x^{-0.4121}$	0.9693	1.4131	0.4121
6–160	$y=0.9539x^{-0.3162}$	0.9859	0.9539	0.3162
6–180	$y=1.3x^{-0.3312}$	0.9739	1.3	0.3312
9–120	$y=1.3307x^{-0.3503}$	0.9869	1.3307	0.3503
9–140	$y=1.157x^{-0.287}$	0.9554	1.157	0.287
9–160	$y=0.6807x^{-0.1319}$	0.9865	0.6807	0.131
9–180	$y=1.4184x^{-0.175}$	0.9782	1.4184	0.175
12–120	$y=1.5192x^{-0.431}$	0.9782	1.5192	0.431
12–140	$y=1.762x^{-0.412}$	0.9861	1.762	0.412
12–160	$y=1.389x^{-0.373}$	0.9713	1.389	0.373
12–180	$y=1.837x^{-0.427}$	0.9725	1.837	0.427

**Table 7:** Performance improvement range of basalt fiber rubber asphalt stress absorption layer.

Performance Index	Fiber Length	Fiber Content	Average Value of Test Data	Increase Range Compared with That without Fiber
Interlaminar shear strength	9 cm	160 g/m^2^	0.496	25.2%
Interlayer bonding strength			0.669	114.4%
Bending fracture energy			20,422.454	305.5%
Total breaking energy			303.405	200.2%

**Table 8:** Design Table for Asphalt Pavement Structure of Lima Expressway in 2022.

Project	Pavement Layer	Pavement Plan	Gate Treatment Plan
		Double Layer Cover Scheme	Milling and Laying Double Layers + Cover Scheme
Pavement Structure Design Drawing	1	Overlay 4 cm SMA-13 (PG76-22 + 3‰ Anti stripping agent)	Overlay 4 cm modified asphalt SMA-13 (PG76-22 + 3‰ Anti stripping agent)
	2	Pave 6 cm modified asphalt high modulus material	Milling and laying back 10 cm modified asphalt Sup-20 (+3‰ basalt fiber)
	3	Rubber asphalt stress absorption layer	Rubber asphalt stress absorption layer
	4	Original surface layer	Original surface layer
Applicable road section		K284 + 398~K286 + 762	Service area runs through lanes and gates

## Data Availability

Data are contained within the article.
